# Evidence-Based Optimal Cutoff Values with the Validation of Criterion-Referenced Standards for Sarcopenic Elderly Fitness Improvement

**DOI:** 10.1155/2020/1638082

**Published:** 2020-04-24

**Authors:** Hyunseok Jee, Soo Hyun Park, Saejong Park

**Affiliations:** ^1^School of Kinesiology, Yeungnam University, 280 Daehak-ro, Gyeongsan, Gyeongbuk 38541, Republic of Korea; ^2^Department of Sport Science, Korea Institute of Sport Science, Seoul, Republic of Korea

## Abstract

This study provides a newly updated perspective of information on severely screened 21 previous studies of the various measurement methods for improving physical fitness and providing determined cutoff values from our reserved elderly human database. We aimed to provide scientific evidence-based information regarding physical fitness standards for developing useful prognostics, promoting and maintaining health programs for sarcopenic elderly. 21 previous studies emphasizing criterion referenced standards and receiver operator characteristic (ROC) curve analyses for improving physical fitness were screened. For predicting the prevalence of sarcopenia, the *t*-test, logistic regression, linear regression, ROC curve analyses, and voluntary categorizations such as the twentieth or sixtieth percentile classification were used. Based on these scientific evidences, we determined cutoff values from our reserved DB and realized that 75 years for men and 70 years for women are the transitional period during which there are large declines in muscle and fat mass (*p* < 0.01), which reflects physical function tests (*p* < 0.01) in both genders. Using the six factors with ideal cutoff thresholds, an individual exercise program can be designed for alleviating symptoms of frailty caused by sarcopenia for the elderly.

## 1. Introduction

Sarcopenia is a progressive deterioration of the loss of muscular mass and strength during aging [1, 2]. Sarcopenia is significantly associated with declining physical activities, which induces a lower level of quality of life that eventually contributes to death. According to the European Working Group on Sarcopenia in Older People (EWGSOP), prognostic criteria for considering the treatment of sarcopenia include muscle mass (quantitative aspect), muscle strength (qualitative aspect), and physical performance [3, 4]. Effective interventions for ameliorating this syndrome have been considered, from pharmaceutical to nutritional and physical training aspects, to decelerate the gradual onset of the sarcopenic state. Jung et al. reported that a recommended diet may be associated with higher muscle mass function in Korean elderly people [5], and a traditional British dietary pattern is associated with a 2.1-fold increase in the risk of prevalent sarcopenia, even with adequate protein intake [6]. About 10% of community-dwelling older adults and 35% of them under institutional care fail to eat enough recommended daily protein intake (0.7 g/kg body weight/day) [7]. Especially, this study is focused on physical activities, which appear to be less adverse-effective than other methods and even lead to positively broad-ranging effects, not only on physical but also on psychological aspects of our bodies. Physical fitness is an important and prerequisite condition for the suitable exercise treatment as an intervention for decelerating the progression of sarcopenia, at the very least.

An inappropriate physical fitness level by way of physical inactivity can result in metabolic chronic diseases such as high blood pressure and diabetes. Declining physical fitness-related factors according to aging are raised, such as physiological function (muscle strength, balance, weight, grip strength, and BMI), fatigue, depression, and cognitive recognition, and improving these factors should be primarily considered the main standard for developing effective intervention to promote physical fitness in the elderly [8, 9]. A series of studies on considering the supportive concepts of “exercise as medicine” demonstrated that regular physical exercise can protect against metabolic disease, cancer, retinal degeneration, and even memory loss [10].

In elderly participants with nonphysical activity, their health state can be easily aggravated via various vulnerabilities caused by the lack of physical fitness, including derived chronic diseases, which can turn into long-lasting frailty. Aging muscle mass decreases by about 2–3% every year after 60 years of age, which causes frailty (expressed as sarcopenia or declining physiological reservoir) as one of the geriatric syndromes due to decreased functional abilities (e.g., muscle strength, walking speed, and balance) [11, 12]. Fried et al. define frailty as characterized by the presence of more than three among five specific items: decreased vitality, slower gait speed, weakness, decreased physical activities, and decreased weight [13]. Prognostic categorization defining by pre- and postpathogenesis should be emphasized. Furthermore, the standard defined by the above clear categorization should be included in the notion of frailty in order to develop and provide an optimized frailty prevention program, to help delay the effects of the aging process.

Developing health-related standards for promoting physical fitness in elderly people have not only socioeconomic but also individual-centered benefits for relieving related chronic disease derived from poor lifestyle choices, which provides scientific evidence-based information for pursuing improvements in the quality of life for each individual.

The main aims of the study are detailed as follows: by searching case studies worldwide regarding validated criterion and reference standards for physical fitness and using an elderly cohort physical fitness-related database (DB) established with patients all across Korea, this study traces prevalence patterns of declines in recognition and physical fitness in the elderly population. We thus aimed to provide scientific evidence-based information regarding physical fitness standards for developing useful prognostics and promoting and maintaining a health program through this field study.

## 2. Materials and Methods

### 2.1. The Characteristics of Elderly Subjects

One thousand four hundred and ninety-five elderly subjects were randomly selected for recruiting from the representative countrywide physical fitness DB of elderly population during 2014 to 2015. Eighteen variables were applied to the gender-specific subject subgroups to analyze the characteristics of the population (Table 1). The variables are as follows: age (yrs), height (cm), weight (kg), body mass index (BMI, kg/m^2^), lean body mass (kg), % body fat (%), waist (cm), total cholesterol (TC, mg/dl), triglycerides (TG, mg/dl), HDL-C (mg/dl), LDL-C (mg/dl), serum glucose (mg/dl), systolic blood pressure (SBP, mmHg), diastolic blood pressure (DBP, mmHg), arm curl (repetition), grip (kg), low limb extension (LLE, Nm), and low limb flexion (LLF, Nm). This study was approved by the institutional review board at the Korea Institute of Sport Science (KISS-201504-EFS-002-01).

### 2.2. Identification and Selection of Studies

**Figure 1:**
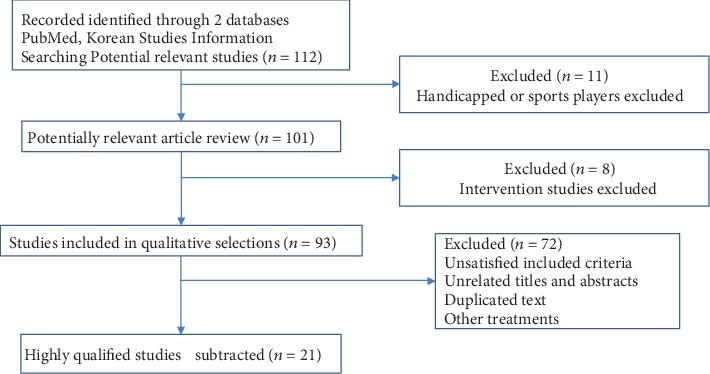
The screening process of physical fitness-relevant appropriate threshold values to identify studies.

Twenty-one previous studies emphasizing criterion referenced standards (CRS) and receiver operator characteristic (ROC) curve analyses for improving physical fitness are shown in Table 2. The severe screening process to identify the final studies selected involved a literature search in two databases, PubMed and the Research Information Service System (RISS, https://www.riss.kr/index.do; *n* = 112). Relevant articles were reviewed; until finally, 21 studies were selected in accordance with our chosen criteria for subjects, including criteria, title, abstract, and treatment (Figure 1). Optimized variables such as physiological and physical function-related variables and statistical methods were examined to apply to further our field study.

This study describes the study elimination process. Reasonable thresholds for physical fitness are pursued by various methods. Potentially related previous studies were identified through PubMed and the Research Information Service System (RISS, https://www.riss.kr/index.do). Three severe screening stages, excluding such unrelated with the scope, allow subtracting to obtain the highly qualified 21 studies.

### 2.3. ROC-Related Outcomes from Elderly Subjects as a Field Study

The main outcome predicting sarcopenia prevalence is measured in both genders in over 70 years of age population. We incorporated the definition of sarcopenia according to the indication from the recommendation of the European Working Group on Sarcopenia in Older People (EWGSOP) [3]. The measured profiles for indicating ideal thresholds are as follows: arm curl (30-second arm curl, number of biceps curls that can be completed in 30 seconds holding a hand weight of five pounds, which was 2.3 kg, for women, and eight pounds, which is 3.6 kg, for men) and low limb strength extension (Nm, lower limb extension force was measured (AP3150, Hur, Kokkola, Finland)). The maximal force output from two trials was measured at 0.01 kg units; sit-to-stand (repetition, maximal sit-to-stand number counted within 30 seconds), timed up and go test (TUG, in seconds, the time to stand from a chair, walk three meters, and walk back and sit down in the chair), and six-minute walk (m, walking distance for six minutes).

### 2.4. Statistics

Whole data were described as the mean ± standard deviation (S.D.). According to the previous study describing the skeletal muscle mass index (SMI, kg/m^2^), we expressed lower than two standard deviations below the mean value as compared with the SMI value as low muscle and the opposite defined as high muscle [1]. A *t*-test was used to examine the differences between the high and low muscles of each variable in the elderly population (Table 1). For cutoff values, logistic regression involving data from sarcopenic elderly individuals was performed, and each of the significant variables showed a difference in cutoff threshold with respect to gender (Table 3). SAS version 9.4 (SAS Institute, Cary, NC, USA) and SPSS version 18.0 (IBM, Armonk, NY, USA) were used for all statistical analyses. A value of *p* < 0.05 was considered to indicate statistically significant difference in all analyses.

## 3. Results

### 3.1. The Characteristics of Elderly Subjects

**Figure 2:**
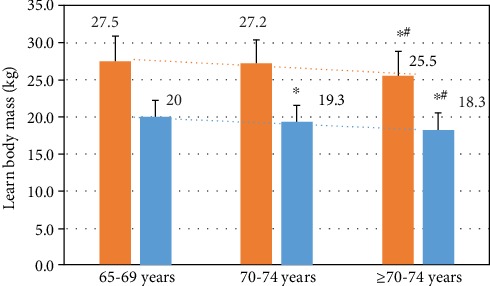
Lean body mass according to different aging groups.

In our DB, 75 years for men and 70 years for women were determined to be the transitional age dividing high and low muscle compositions in both genders (*p* < 0.01) (Figure 2). An abrupt decrease in muscle mass with rapidly increasing fat mass is shown in this age period; BMI, lean mass, per BF (*p* < 0.01); however, physiological variables such as total cholesterol (TC), triglyceride (TG), high-density lipoprotein (HDL), low-density lipoprotein (LDL), serum glucose, and blood pressure (BP) do not significantly change in this age period. Physical function tests show all significant differences that were present in the high muscle mass group in both genders have significantly higher values as compared with those in the lower muscle mass group (*p* < 0.01) (Table 1).

Three aging groups such as 65-69, 70-74, and more than 75 years are sex-differently divided according to the value (kg) of the lean body mass. Orange and blue indicate the men and women groups, respectively. ∗ indicates significant difference (*p* < 0.05) to 65-69 year group of corresponding sex; # indicates significant difference (*p* < 0.05) to each sex group of 70-74 years.

### 3.2. 21 Selected Studies

In selecting highly qualified articles relevant to ROC for physical fitness, 21 articles were finally selected and are shown in Table 2. In the articles, measuring methods are mainly focused on various ranges of age as a means to move forward for the next analysis as this field study. Target variables for CRS are categorized as physiological (e.g., BP, TG, HDL, glucose, insulin, BMI, VO_2_ max, and cholesterol) and physical function (e.g., muscle strength, physical compositions, and walking and running tests) factors. Logistic regression, ROC curve analysis, linear regressions, validation methods, and voluntary categorization such as the 20^th^ or 60^th^ percentile classification were examined. Other factors such as ethnical differences and specific ranges of age are overlooked for the further study.

### 3.3. Physical Fitness-Related ROC Curve Outcomes for Preventing Prognostic Sarcopenia

In gender-different elderly populations, ROC curve analysis for setting optimal cutoff thresholds with ideal sensitivity and specificity values provides priceless information for developing interventions for ameliorating sarcopenia symptoms (Table 3). When predicting prevalence of sarcopenia in elderly people, six physical functions (arm curl, limb strength extension and flexion, sit-to-stand, TUG, and six-minute walk test) in both genders all have significant *p* values (*p* < 0.05). The cutoff values of sit-to-stand and six-minute walk tests in elderly women have better thresholds than those in elderly men. Sensitivity of low limb strength extension and flexion is 83% and 80% in men and 86% and 86% in women, respectively. Specificity of the six-minute walk test in men and women is 93% and 82%, respectively. Comparatively high values in sensitivity and specificity (both with more than 70%) are shown in low limb strength extension (83% and 75%), low limb strength flexion (80% and 76%), and TUG (71% and 70%) only in men.

## 4. Discussion

In this study, we searched 21 severely screened previous studies to identify the measurement methods regarding improving physical fitness without consideration of specific age periods. Logistic regression, ROC curve analysis, linear regression, and voluntary categorizations such as the twentieth or sixtieth percentile classifications were used to provide cutoff points of physical fitness profiles. Based on this scientific evidence, we determined cutoff threshold values from our reserved elderly human DB and realized that 75 years for men and 70 years for women are the transitional age period for muscle and fat mass (*p* < 0.01), which reflects physical function tests (*p* < 0.01) that indicate physical fitness levels in both genders.

### 4.1. Why This Study Focuses on Elderly People and Provides Information That Allows to Develop Useful Prognostics, Maintaining and Promoting Health Programs

Our study for setting health-related CRS focuses on elderly individuals who especially have limitations in their physical abilities. Factors directly related to metabolic disorder are remarkably relevant to physical fitness factors. Comparative hand grip strength, seated hip adductor stretching, and six-minute walk test in men and comparative hand grip strength in women significantly affect as physical factors on classifying physical fitness in elderly peoples as the prefrailty and frailty. The most important factor affecting frailty in the aged is comparative hand grip strength. Individuals with lower comparative grip strength (i.e., the lower 33.3% group) present with three times more chronic prevalence diseases as compared with those with higher comparative grip strength (i.e., the higher 33.3% group). Elderly individuals with comparative higher grip strength are also typically confident in recognition and balance capacity (https://nfa.kspo.or.kr). In light of these results, it should be noted that the elderly population in Korea represented around 12.2% of the total population in 2013 and will be more than 24% in 2030 (https://kostat.go.kr/portal/eng/index.action). Thus, it is important to provide beneficial information to elongate the healthy lifespan as much as possible.

### 4.2. ROC and Non-ROC-Used Methods or Analysis of Criterion-Referenced Standards (CRS) by Selected Articles

Research efforts for establishing CRS and ROC regarding physical fitness for ameliorating and at least maintaining a healthy state have been made, and some studies were thus selected through a severe screening process in this study to identify the chosen variables in various age groups, with an aim at providing evidence-based information to design a preventive and prognostic sarcopenic management program for elderly people by incorporating appropriate ROC values from our DB, as we clarified the aim of this study in Introduction for this field study. One study used height, BMI, waist, and fat ratio as dependent variables with a 20 m shuttle run as an independent variable for oxidative capacity for comparing overweight kids with normal kids to decide the minimal cutoff point for oxidative capacity in children [14]. A prognostic study for preventing the risk of cardiovascular diseases in kids according to cutoff points of metabolic risk provided each of suitable thresholds by using quartile [15]. Another study used the ROC curve to determine the cutoff threshold of each physical composition regarding the risky relationship between type II diabetes and cardiovascular diseases in American football players [16].

A nine-year follow-up study provided cutoff points by using the ROC curve for a threshold between muscular strength and metabolic syndrome in aging men (5,685 under 50 years vs. 1,541 over 50 years of age) [17]. In this study, three study groups were divided as model 1 (no exercise during the last three months), model 2 (middle level of exercise during the last three months), and model 3 (vigorous level of exercise during the last three months).

Another study provided a risk threshold of physiological factors (VO2 max, HDL, glucose, and triglyceride) in oxidative capacity via using the ROC curve for age and gender-differentiated adolescents between 12~18 yrs (*n* = 1240), in order to show the reasonable area of the risk threshold at each one-year point to prevent metabolic disease [18].

Other studies used cross-validation for a more accurate cutoff point, rather than ROC curve analysis [19]. Hooten et al. used multiple variable linear regression for the relation between knee joint kinetics and pressure pain threshold [20]. Multiple regression was used for predictive factors such as physical composition to physical fitness standards [21]. Gorman et al. used logistic regression [22], while Duncan et al. randomly divided groups into the following quantile ranks according to cardiorespiratory fitness: lower, middle, and high groups, representing the lower twentieth, twentieth to fifty-ninth, and over sixtieth, respectively [23]. A fourteen-year follow-up longitudinal study for 266 type 2 diabetic patients shows that the symptoms of type 2 diabetes in 33% declined due to the promotion of a physical fitness program [24]. The criterion reference standard for physical activity used in this study was baseline VO2 max at an AUC of 0.70 (95% CI, 0.66-0.73, with sensitivity and specificity being 0.64, respectively) and the optimal cutoff threshold was decided as 10.8 METs for cardiorespiratory fitness.

### 4.3. Providing Cutoff Threshold for Diagnostic and Preventive Sarcopenia

Based on the screened previous studies, we decided to provide the cutoff points of meaningful values from our own elderly human DB. Six physically functional profiles are disclosed to have significant intereffects with sarcopenia in both genders (*p* < 0.05), and are as follows: arm curl, low limb strength extension, low limb strength flexion, sit-to-stand, TUG, and six-minute walk test. Absolute muscle output force from the upper and lower limbs is likely higher in men; however, such results should be considered together with the ratio with their burdening load, such as weight. It cannot then possibly determine that necessary muscle force is sufficient for their physical functionality. The sit-to-stand and six-minute walk tests indicate all physical functions necessary for resistant and oxidative capacities, and elderly women have higher values in these tests. For longevity, muscular oxidative capacity is possibly required more so than other capacities (of note, the longevity of Korean women ranked third in the world in 2015). In relation with muscular mass comparison between high and low muscles, the average age difference is also shown to be 70 and 73 years, respectively (Table 1). This suggests that this is a decisive turning point of age on the abrupt muscular declines in both qualitative and quantitative ways. Using the six factors with ideal cutoff thresholds can be used to effectively design a tailor-made individual exercise program for alleviating the worsen symptom of frailty caused by sarcopenia for the decisive period of elderly.

## 5. Conclusions

To provide evidence-based scientific information for useful prognostics and for promoting and maintaining a health program, our study identified beneficial outcomes, like those in the following:

1. We pinpointed 21 relevant severely selected articles that include logistic regression, ROC curve analysis, linear regression, and voluntary categorizations to produce optimized cutoff points of physical fitness profiles
2. We continuously traced ideal cutoff threshold values from our reserved elderly human DB and found that individuals aged from 75 years for men and 70 years for women are within the specifically transitional age period for muscle mass (*p* < 0.01), which reflects the need for physical function tests in both genders (*p* < 0.01)

Worsened symptom of sarcopenia usually induces fall in aged persons because of decreased quality and quantity of muscle, which is directly related to the low quality of life in elderly peoples. As the prognostic cutoff values of each physical fitness-related variable for the aged person were provided, ideal physical fitness promoting program can be designed and applied to promote muscle function or at least deaccelerate the worsening of muscle function which possibly contributes to the independent life of aged peoples. This evidence-based field study can contribute to strengthen the standardization of physical fitness-related variables, and the benefit of lifelong health promotion in senescent elderly peoples and the results in this study can be used for a health policy to prevent the symptom of sarcopenia by publicizing as a subject of national-wide recommendation.

Regular exercise elderly-doers decrease possibility to be hospitalized, which induces successful aging such as better quality of life and independent life. An optimal socioeconomic model can be suggested through which each aged person lives a healthy life by reducing medical expenses via doing exercise with a reasonably designed exercise program.

## Figures and Tables

**Table 1:** The characteristics of elderly subjects.

	Men (*n* = 585)			Women (*n* = 910)		
	High muscle (*n* = 397)	Low muscle (*n* = 188)	*p* value	High muscle (*n* = 618)	Low muscle (*n* = 292)	*p* value
Age (yr)	70.92 ± 4.36	73.12 ± 5.13	<0.001	70.89 ± 4.91	73.94 ± 5.54	<0.001
High (cm)	167.67 ± 4.95	161.45 ± 4.78	<0.001	154.14 ± 4.29	148.19 ± 4.22	<0.001
Weight (kg)	68.74 ± 6.64	57.80 ± 7.19	<0.001	60.24 ± 7.06	50.74 ± 5.57	<0.001
BMI (kg/m^2^)	24.48 ± 2.21	22.19 ± 2.45	<0.001	25.36 ± 3.13	23.16 ± 2.58	<0.001
Lean mass (kg)	28.60 ± 2.40	23.31 ± 1.88	<0.001	20.47 ± 1.62	16.80 ± 1.19	<0.001
Per BF (%)	24.53 ± 5.03	24.44 ± 6.30	0.855	35.64 ± 6.49	35.91 ± 5.74	0.524
Waist (cm)	88.30 ± 6.45	82.17 ± 7.40	<0.001	89.53 ± 8.77	84.10 ± 8.34	<0.001
TC (mg/dl)	184.78 ± 35.59	180.61 ± 36.63	0.209	195.88 ± 37.52	196.70 ± 36.00	0.762
TG (mg/dl)	109.29 ± 49.16	98.99 ± 40.98	0.012	118.51 ± 49.67	123.53 ± 55.50	0.187
HDL-C (mg/dl)	54.85 ± 12.90	56.68 ± 13.73	0.133	57.80 ± 14.05	58.28 ± 14.07	0.635
LDL-C (mg/dl)	117.40 ± 32.67	112.13 ± 31.78	0.077	123.32 ± 34.23	122.81 ± 32.61	0.835
Glucose (mg/dl)	101.08 ± 16.50	98.73 ± 17.02	0.128	99.43 ± 17.32	98.76 ± 19.13	0.614
SBP (mmHg)	129.39 ± 14.78	129.54 ± 15.57	0.910	130.78 ± 16.48	131.25 ± 17.44	0.692
DBP (mmHg)	74.58 ± 9.07	74.09 ± 9.43	0.554	74.61 ± 9.36	73.27 ± 9.24	0.045
Arm curl (rep)	20.06 ± 5.13	17.03 ± 4.78	<0.001	17.03 ± 4.45	15.13 ± 4.18	<0.001
Grip (kg)	36.11 ± 5.27	30.47 ± 4.71	<0.001	22.79 ± 3.90	19.58 ± 3.29	<0.001
Low limb extension (nm)	141.95 ± 36.75	108.99 ± 26.26	<0.001	83.15 ± 21.93	68.28 ± 18.43	<0.001
Low limb flexion (nm)	81.15 ± 21.75	60.61 ± 17.01	<0.001	45.50 ± 13.73	34.80 ± 10.40	<0.001

**Table 2:** Screened studies using various methods for measuring physical fitness.

Authors	Subjects	Target for criterion referenced standards (CRS)	Methods of criterion-referenced standards	Miscellaneous
Amini et al. [11]	American football players (*n* = 62)	Waist circumference, quadriceps leak torque, systolic & diastolic BP	ROC curve analysis followed by logistic regression for prediction	Lessening cardiometabolic risk-like type 2 diabetes and cardiovascular disease
Jang [12]	Pre & post 50 yrs men (*n* = 7226)	Muscle strength and metabolic syndrome (triglyceride, HDL cholesterol, glucose, systolic diastolic BP)	ROC and 20% below −> low level and logistic regression (low muscle strength vs. metabolic syndrome)	Low muscle strength: lowest age-specific 20^th^ percentile (2.56 kg/kg body weight in pre 50 vs. 2.50 kg/kg body weight in post 50) High muscle strength: highest age-specific 20^th^ percentile
Ruiz et al. [15]	69 pain treatment patients	Lower pressure pain threshold (PPT, dependent), knee mechanics (independent)	Univariable linear regression for lower PPT and multiple variable linear regression	Knee mechanics are associated with PPT
Sénéchal et al. [17]	Systematic review (*n* = 59)	Moderate to vigorous physical activity, 6MWT	Logistic regression for cutoff (accelerometry measured moderate to vigorous physical activity compared to sedentary in elderly) and Bland-Altman method used; however, the results are obscure	Sedentary ratio, activity difference, and exercise time reflect the results assessed by accelerometry
Hooten et al. [20]	Review			Changed evaluation trends shown from performance centered to health-related test and norm referenced to CR evaluation. Criterion-referenced standards vs. norm-referenced evaluation ROC introduced
Hanifah et al. [21]	Review			Criterion-referenced evaluation developed history. Advantages of CRS (absolute, diagnostic supportive), drawbacks of CRS (misclassification such as false mastery and false nonmastery, nonsufficient incentives etc.)

Authors	Subjects	Target for criterion-referenced standards (CRS)	Methods of criterion-referenced standards	Miscellaneous
Gorman et al. [22]	65~84 yrs both gender elderly (*n* = 3074)	Chair stands in 30 s, arm curls in 30 s, 6 min walk, 8 foot up and go (1)	ROC for predicting independent physical function in later life and logistic regression	For later life (~90 yrs), internet-based program recommended (https://www.fmh.ulisboa.pt/ehlab/calculator/)
Duncan et al. [23]	Review (setting CRS transition history)	Make participants get into HFZ	ROC analysis, LMS (*L* = skewness, *M* = median, *S* = coefficient of variation), centile	Single cutoff score ≥ health fitness zone (HFZ), needs improvement zone (NIZ) set for warning potential risk
Kawakami et al. [24]	Systematic review			Selected article had different methods, analyses, and results that prevented comparison between studies ≥ junk article
Zhu et al. [25]	55 yrs and older both gender elderly (*n* = 3392)	BMI, hand grip strength	ROC for hand grip strength cut-points and impaired hand grip cut-points vs. mobility limitation by logistic regression	Optimal hand grip strength cut-points for mobility limitation and the cut-points discriminate BMI
Cureton and Warren [26]	Systematic review		False-positive/false-negative analysis, regression model, visual inspection	

**Table 3:** Results of ROC curve analysis predicting prevalence sarcopenia in elderly.

Senior fitness variables	Prevalence (%)	AUC (95% CI)	Cutoff value	Sensitivity	Specificity	*p* value
Men						
Arm curl (rep.)	8.45	0.718 (0.679~0.754)	≤16	68.75	71.54	<0.001
Low limb strength_ extension (nm)	7.90	0.849 (0.815~0.879)	≤109.22	82.93	75.31	<0.001
Low limb strength_ flexion (nm)	7.90	0.829 (0.794∼0.861)	≤61.72	80.49	75.94	<0.001
Sit-to-stand (rep.)	7.50	0.714 (0.674∼0.753)	≤12	48.72	83.58	<0.001
TUG (sec.)	7.61	0.763 (0.725∼0.798)	>6.367	71.43	70.00	<0.001
6 min walk (m)	6.51	0.707 (0.655∼0.755)	≤388.3	40.91	93.35	0.019
Women						
Arm curl (rep.)	12.47	0.715 (0.684~0.744)	≤14	60.36	71.50	<0.001
Low limb strength extension (nm)	9.97	0.728 (0.695~0.760)	≤76.04	86.49	56.74	<0.001
Low limb strength flexion (nm)	9.97	0.793 (0.762∼0.821)	≤37.74	86.49	64.67	<0.001
Sit-to-stand (rep.)	12.0	0.676 (0.641∼0.710)	≤14	69.66	61.81	<0.001
TUG (sec.)	11.9	0.702 (0.669∼0.733)	>6.687	68.37	61.29	<0.001
6 min walk (m)	7.46	0.793 (0.746∼0.835)	≤402.8	68.00	82.90	0.019

## Data Availability

The data used to support the findings of this study have been deposited in the Korea Institute of Sport Science.
